# Molecular Effects of Auto-Antibodies on Angiotensin II Type 1 Receptor Signaling and Cell Proliferation

**DOI:** 10.3390/ijms23073984

**Published:** 2022-04-02

**Authors:** Aurélie Philippe, Gunnar Kleinau, Jason Jannis Gruner, Sumin Wu, Daniel Postpieszala, David Speck, Harald Heidecke, Simon J. Dowell, Gabriela Riemekasten, Peter W. Hildebrand, Julian Kamhieh-Milz, Rusan Catar, Michal Szczepek, Duska Dragun, Patrick Scheerer

**Affiliations:** 1 Berlin Institute of Health at Charité—Universitätsmedizin Berlin, BIH Biomedical Innovation Academy, D-10178 Berlin, Germany; 2 Charité—Universitätsmedizin Berlin, Corporate Member of Freie Universität Berlin and Humboldt-Universität zu Berlin, Department of Nephrology and Medical Intensive Care, Campus Virchow Klinikum, D-13353 Berlin, Germany; jannis.gruner@vivantes.de (J.J.G.); wusumin89@163.com (S.W.); d.postpieszala@googlemail.com (D.P.); rusan.catar@charite.de (R.C.); 3 Charité—Universitätsmedizin Berlin, Corporate Member of Freie Universität Berlin and Humboldt-Universität zu Berlin, Center for Cardiovascular Research, D-10117 Berlin, Germany; 4 Charité—Universitätsmedizin Berlin, Corporate Member of Freie Universität Berlin and Humboldt-Universität zu Berlin, Institute of Medical Physics and Biophysics, Group Protein X-ray Crystallography and Signal Transduction, D-10117 Berlin, Germany; gunnar.kleinau@charite.de (G.K.); david.speck@charite.de (D.S.); peter.hildebrand@medizin.uni-leipzig.de (P.W.H.); michal.szczepek@charite.de (M.S.); 5 Vivantes Humboldt-Klinikum, Department of Urology, D-13509 Berlin, Germany; 6 CellTrend GmbH, D-14943 Luckenwalde, Germany; heidecke@celltrend.de; 7 GlaxoSmithKline, Stevenage SG1 2NY, UK; simon.j.dowell@gsk.com; 8 Priority Area Asthma & Allergy, Research Center Borstel, Airway Research Center North (ARCN), Members of the German Center for Lung Research (DZL), D-23845 Borstel, Germany; gabriela.riemekasten@uksh.de; 9 University of Lübeck, University Clinic Schleswig-Holstein, Department of Rheumatology and Clinical Immunology, Campus Lübeck, D-23538 Lübeck, Germany; 10 Leipzig University, Medical Faculty Leipzig, Institute for Medical Physics and Biophysics, D-04107 Leipzig, Germany; 11 Berlin Institute of Health at Charité—Universitätsmedizin Berlin, D-10178 Berlin, Germany; 12 Charité—Universitätsmedizin Berlin, Corporate Member of Freie Universität Berlin and Humboldt-Universität zu Berlin, Department of Transfusion Medicine, D-10117 Berlin, Germany; julian.milz@charite.de; 13 DZHK (German Centre for Cardiovascular Research), Partner Site Berlin, D-13353 Berlin, Germany

**Keywords:** angiotensin II type 1 receptor, AT_1_R, auto-antibodies, G protein-coupled receptors, systemic sclerosis, angiotensin, endothelin

## Abstract

The angiotensin II (Ang II) type 1 receptor (AT_1_R) is involved in the regulation of blood pressure (through vasoconstriction) and water and ion homeostasis (mediated by interaction with the endogenous agonist). AT_1_R can also be activated by auto-antibodies (AT_1_R-Abs), which are associated with manifold diseases, such as obliterative vasculopathy, preeclampsia and systemic sclerosis. Knowledge of the molecular mechanisms related to AT_1_R-Abs binding and associated signaling cascade (dys-)regulation remains fragmentary. The goal of this study was, therefore, to investigate details of the effects of AT_1_R-Abs on G-protein signaling and subsequent cell proliferation, as well as the putative contribution of the three extracellular receptor loops (ELs) to Abs-AT_1_R signaling. AT_1_R-Abs induced nuclear factor of activated T-cells (NFAT) signaling, which reflects G_q/11_ and G_i_ activation. The impact on cell proliferation was tested in different cell systems, as well as activation-triggered receptor internalization. Blockwise alanine substitutions were designed to potentially investigate the role of ELs in AT_1_R-Abs-mediated effects. First, we demonstrate that Ang II-mediated internalization of AT_1_R is impeded by binding of AT_1_R-Abs. Secondly, exclusive AT_1_R-Abs-induced G_q/11_ activation is most significant for NFAT stimulation and mediates cell proliferation. Interestingly, our studies also reveal that ligand-independent, baseline AT_1_R activation of G_i_ signaling has, in turn, a negative effect on cell proliferation. Indeed, inhibition of G_i_ basal activity potentiates proliferation triggered by AT_1_R-Abs. Finally, although AT_1_R containing EL1 and EL3 blockwise alanine mutations were not expressed on the human embryonic kidney293T (HEK293T) cell surface, we at least confirmed that parts of EL2 are involved in interactions between AT_1_R and Abs. This current study thus provides extended insights into the molecular action of AT_1_R-Abs and associated mechanisms of interrelated pathogenesis.

## 1. Introduction

The superfamily of G protein-coupled receptors (GPCRs) constitutes the largest membrane-spanning protein family in the human genome. More than 800 different human GPCRs transmit a huge variety of extracellular signals into the cytoplasm. Activating stimuli include peptides, neurotransmitters, chemokines, ions, metabolites, fatty acids, and even physical stimuli, such as light, mechanical forces, and pH shifts [1,2].

Angiotensin II type 1 receptor (AT_1_R) is a class A GPCR endogenously activated by angiotensin II (Ang II) and is evolutionarily related to angiotensin II type 2 receptor (AT_2_R). AT_1_R is expressed in the kidneys, adrenal gland, nervous system, heart and blood vessels [3]. Stimulation of AT_1_R results in the intracellular recruitment of various effectors, but especially in activation of G_q/11_ [4,5,6,7], which triggers, e.g., the Extracellular signal-Regulated Kinases 1/2 (ERK1/2) signaling pathway, thus driving blood pressure regulation via vasoconstriction, or water and ion homeostasis [8,9].

In 1999, auto-antibodies directed against AT_1_R (AT_1_R-Abs) were identified in women afflicted with preeclampsia [10]. Several further studies confirmed that AT_1_R autoantibodies act as agonistic modulators triggering various pathogenic conditions [11,12,13]. Auto-antibodies have been identified in patients with acute vascular graft rejection, triggering the pathogenic activation of Nuclear Factor of “κappa-light-chain-enhancer” of activated B-cells (NFκB) and Activator Protein 1 (AP1), contributing to obliterative vasculopathy [14,15,16]. The association of AT_1_R-Abs with clinical features has also been extensively studied and demonstrated in the context of transplantations [17,18,19,20,21], as well as in preeclampsia, where it affects angiogenesis [22,23,24].

Binding of AT_1_R-Abs promotes downstream signaling through the activation of AT_1_R [14,25,26]. While Ang II receptor binding has been explored in detail [27,28,29,30,31], and a receptor–ligand complex structure (PDB ID: 6oS0 [32]) is known, the detailed molecular mechanisms of the antibody–receptor interaction and associated effects are still unknown, hampering a comprehensive understanding of AT_1_R-Abs-related pathogenesis at the molecular level.

Of particular note is a newly recognized link between COVID-19 infection and Ang II-AT_1_R signaling, which is involved in inflammatory processes, collateral tissue damage, and systemic failure [33]. Such viral infection dysregulates renin–angiotensin–aldosterone system (RAAS) homeostasis by elevating Ang II levels. AT_1_R blockers or biased AT_1_R agonists are considered to potentially complement COVID-19 treatment strategies, including severe acute respiratory syndrome coronavirus-2 (SARS-CoV-2) [34,35,36].

Angiotensin Converting Enzyme inhibitors (ACEis) are the drugs currently recommended to treat scleroderma renal crisis (SRC) [37] and have been found to result in a substantial improvement in the prognosis of this pathogenic condition [38]. However, recent studies have questioned the use of this drug because it was shown that ACEis are associated with a shorter time to occurrence of vascular lesions in systemic sclerosis (SSc) patients [39]. In a retrospective study, it was found that development of SRC was linked to the prescription of ACEis [40]. In addition, an analysis of EULAR registers unraveled that ACEi treatment was an independent risk factor for SRC [41]. On the contrary, Angiotensin Receptor Blockers (ARB) were not associated with any risks in these studies. Hence, better understanding of the activation of AT_1_R after stimulation with its natural ligand Ang II or patient antibodies could hold the key to developing innovative therapeutic strategies to prevent the occurrence of and treat SRC.

We elucidated here the impact of AT_1_R-Abs in SSc patients on the AT_1_R-mediated signaling in endothelial cells. In addition, we aimed to investigate the influence of the three AT_1_R extracellular loops (ELs) on autoantibody-induced receptor activation by substituting the sequence of AT_1_R ELs with alanine amino acid blocks and thereby delineate and further describe the determinants and regions mediating the effects of AT_1_R Abs.

The goal of this study was to provide further information about potential targets for pharmacological intervention strategies in AT_1_R-related signaling.

## 2. Results

### 2.1. Endothelial Cell Proliferation Is Induced by AT_1_R-Abs-Mediated G_q/11_ Activation and Is Inhibited by Basal G_i_ Signaling

Ang II is known to activate G-proteins G_i_ and G_q/11_ intracellularly [42]. The latter induces phospholipase C activation and increases cytosolic Ca^2+^ concentration, which, in turn, triggers cellular responses, such as protein kinase C stimulation and nuclear factor of activated T-cells (NFAT) signaling [43], which we measured here as a signaling readout.

Moreover, G_q/11_ and G_i_ activation have also been reported to be potentially involved in the proliferation of specific cell types [42,44,45,46]—a key mechanism in obliterative vasculopathy. Therefore, we assessed NFAT signaling pathway stimulation by AT_1_R-Abs and the impact of such signaling on endothelial cell proliferation. For this purpose, we isolated AT_1_R-Abs from SSc patients with renal crisis. Of note, these specific SSc patients carried AT_1_R-Abs as well as antibodies targeting another class A GPCR, the endothelin-1 type A receptor (ET_A_R). To focus on AT_1_R-mediated effects, we used Human Microvascular Endothelial Cells (HMEC-1s), which endogenously express angiotensin receptors (ATRs) and ET_A_R. In the non-transfected state, HMEC-1s already show a higher expression of *AGTR1* compared with *EDNRA* (Supplementary Materials Figure S1A), but we additionally transiently transfected these cells to over-express the human wild type receptor (WT AT_1_R), which finally leads to a 10,000-fold increase in *AGTR1* mRNA relative to *EDNRA* mRNA (Supplementary Materials Figure S1B). This over-expression ensured the relative significance of induced effects at AT_1_R above potential effects of ET_A_R in interplay with Abs.

**Figure 1:**
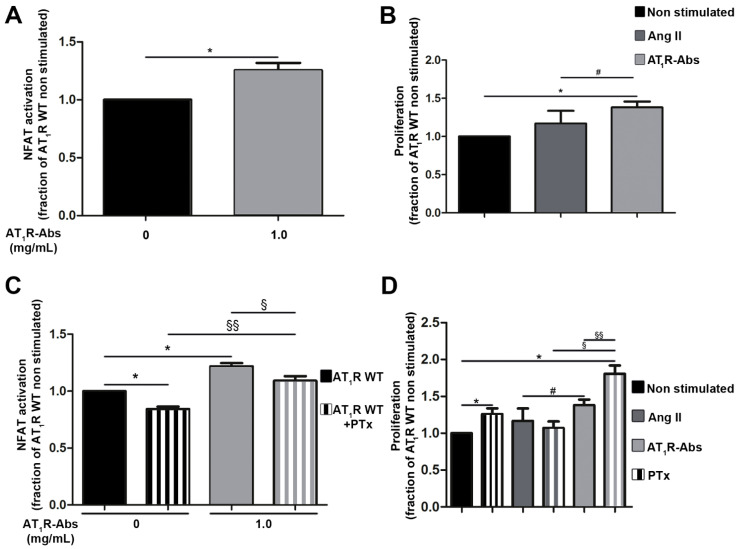
Angiotensin II (Ang II) type 1 receptor auto-antibodies (AT_1_R-Abs)-mediated G_q/11_ activation and induction of human microvascular endothelial cells (HMEC-1) proliferation. (**A**) In HMEC-1s, AT_1_R-Abs stimulation triggered luciferase production mediated by nuclear factor of activated T-cells (NFAT). (**B**) AT_1_R-Abs stimulation but not Ang II (1 µM) stimulation led to cell proliferation. (**C**) One hour pre-incubation with pertussis toxin (PTx) did not change NFAT activation or (**D**) cell proliferation induced by AT_1_R-Abs. Cells transfected to over-express wild-type (WT) AT_1_R were stimulated with 1 mg/mL AT_1_R-Abs or 1 µM Ang II, as indicated, with or without a pre-incubation with 5 ng/mL PTx for one hour. SEMs derived from five to eight experiments are shown along with *p*-values for Wilcoxon or Mann–Whitney tests. (* *p* < 0.05 as compared with AT_1_R WT without stimulation; # *p* < 0.05 as compared with AT_1_R stimulated with Ang II; § *p* < 0.05, §§ *p* < 0.01 as compared with AT_1_R WT pre-incubated with PTx).

After six hours of stimulation with 1 mg/mL AT_1_R-Abs, NFAT activation levels were increased (~30%) compared with non-stimulated cells (Figure 1A). Moreover, cell proliferation was moderately increased by the addition of AT_1_R-Abs, while Ang II effects remained insignificant (Figure 1B).

To potentially distinguish between the effects of either G_q/11_ or G_i_ in AT_1_R-Abs-induced NFAT stimulation, cells were pre-treated with pertussis toxin (PTx) one hour prior to stimulation. PTx specifically inhibits G_i_ signaling [47] and therefore decreases G_i_-related effects on NFAT stimulation by endogenous ligands. Of note, pre-incubation with PTx significantly reduced even basal level NFAT activation (non-stimulated cells, Figure 1C), which indicates G_i_-induced NFAT activation independently of AT_1_R-Abs or Ang II stimulation.

Compared with cells stimulated with AT_1_R-Abs without PTx, NFAT activation was significantly lower in PTx pre-treated cells stimulated with AT_1_R-Abs (Figure 1C). However, the relative decrease in the signaling extending between AT_1_R with or without PTx and between AT_1_R-Abs with or without PTx was similar. Thus, we conclude that G_i_ stimulated by AT_1_R-Abs has no direct effect on the regulation of NFAT signaling. This finding strongly suggests that G_q/11_-mediated signaling through AT_1_R-Abs is a key pathway for NFAT stimulation, while basal G_i_ signaling displays only a minor contribution to the effects on NFAT.

While we showed that AT_1_R-Abs-mediated NFAT signaling increases independently from G_i_, we tested a potential G_i_ involvement in HMEC-1 proliferation by inhibiting G_i_ via PTx in proliferation assays. Surprisingly, we detected a significant increase in cell proliferation when only blocking G_i_ basal activity (non-stimulated cells, Figure 1D), which suggests that basal G_i_ activity reduces cell proliferation. The proliferation-supporting effect triggered upon G_i_ inhibition is further increased in cells pre-treated with PTx and stimulated with AT_1_R-Abs (Figure 1D). Cell proliferation is then doubled due to the combination of G_i_ inhibition and the presence of AT_1_R-Abs, a result in stark contrast to Ang II’s lack of effect on cell proliferation.

In summary, G_i_ inhibition strengthens AT_1_R-Abs-mediated HMEC-1 proliferation (likely mediated by G_q/11_) and points to a “protective” effect of G_i_ towards proliferation mechanisms, an effect which also differs between Ang II and AT_1_R-Abs-induced signaling.

### 2.2. Auto-Antibodies Attenuate Ang II-Mediated Internalization of AT_1_R

These identified AT_1_R-Abs effects on AT_1_R signaling are distinct from Ang II-induced signaling. We next examined the number of receptors expressed at the cell surface; this factor can have major effects on signaling properties [48] and all the more since it is already known that Ang II-mediated AT_1_R signaling capacity depends on internalization [49], whereby, in contrast, a recent study showed that AT_1_R-Abs isolated from preeclamptic women do not induce AT_1_R internalization [50].

**Figure 2:**
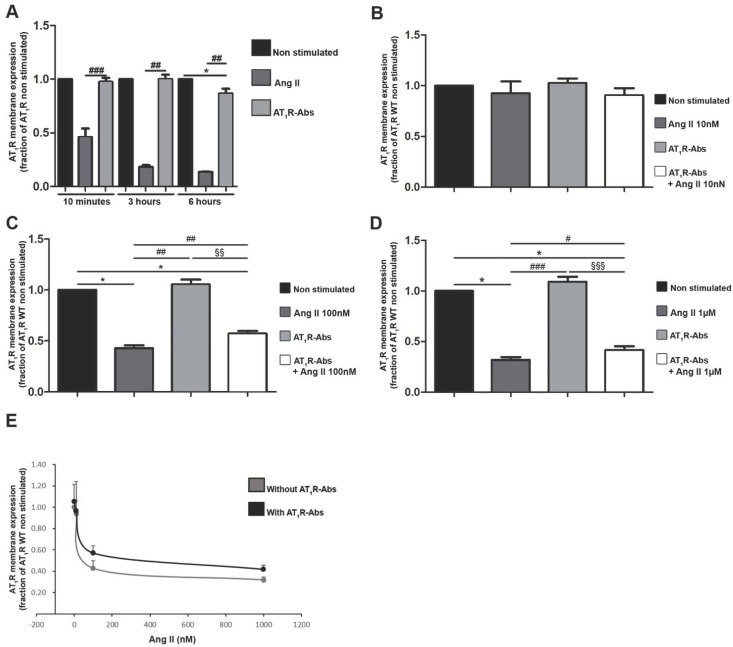
Angiotensin II (Ang II) type 1 receptor auto-antibodies (AT_1_R-Abs)-induced reduction in Ang II-mediated internalization of AT_1_R. (**A**) Human Embryonic Kidney293T cells (HEK293T) transfected to express a HiBiT-tagged AT_1_R were incubated for 10 min, three or six hours with 1 µM Ang II or 1 mg/mL AT_1_R-Abs. (**B**–**D**) Ang II stimulation in HEK293T dose-dependently triggered a decrease in AT_1_R expression at the plasma membrane. Cells transfected with the HiBiT-tagged receptor were pre-incubated with or without 1 mg/mL AT_1_R-Abs for six hours before stimulation with increasing concentrations of Ang II for 10 min. Results are gathered in (**E**). SEMs derived from 7 to 14 experiments (**A**–**D**) are shown along with *p*-values for Wilcoxon or Mann–Whitney tests. (* *p* < 0.05 as compared with HiBiT-AT_1_R WT without stimulation; # *p*<0.05, **##** *p* < 0.01, ### *p* < 0.001 as compared with HiBiT-AT_1_R WT stimulated with Ang II; §§ *p* < 0.01, §§§ *p* < 0.001 as compared with AT_1_R WT pre-incubated with AT_1_R-Abs.)

Short-time incubation of cells with the endogenous AT_1_R ligand Ang II indeed resulted in a decreased number of receptors on the cell surface (Figure 2A), which is in line with the ligand-induced internalization already described [49]. In contrast, ten minutes stimulation with AT_1_R-Abs did not significantly alter AT_1_R numbers at the surface (Figure 2A). This remained true after three hours of stimulation (Figure 2A), while we observed a significant decrease in AT_1_R numbers at the membrane after six hours of stimulation (Figure 2A).

In addition, although effects of AT_1_R-Abs on AT_1_R internalization in the presence of Ang II have not been investigated so far, this condition resembles the endogenous complex situation. We thus investigated whether auto-antibodies could modify Ang II-mediated internalization of the receptor. To address this, Human Embryonic Kidney293T cells (HEK293T) expressing HiBiT-tagged AT_1_Rs were pre-incubated (or not) with AT_1_R-Abs and then stimulated with increasing doses of Ang II (Figure 2B–E). Cells pre-incubated with AT_1_R-Abs and then stimulated with 100 nM or 1 µM Ang II showed significantly higher numbers of AT_1_Rs on the plasma membrane than those that received Ang II stimulation alone (Figure 2C,D). Therefore, it can be concluded that AT_1_R-Abs attenuate Ang II-mediated internalization of the receptors.

### 2.3. The Second Extracellular Loop Is Involved in AT_1_R-Mediated Signaling Induced by AT_1_R Abs

**Figure 3:**
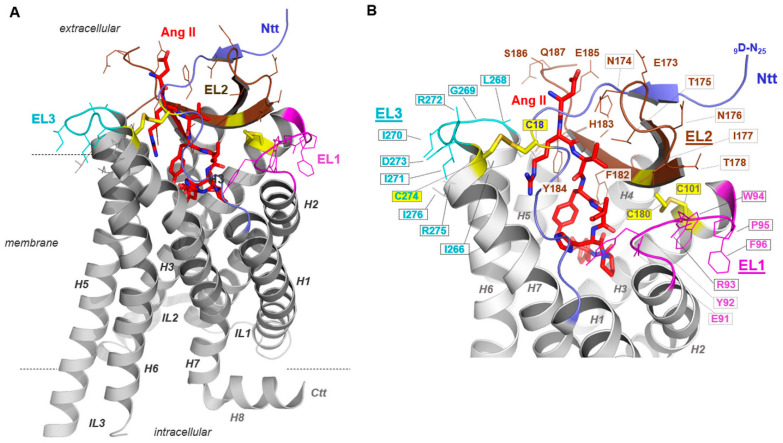
Active Angiotensin II type 1 receptor (AT_1_R) conformation bound with angiotensin II (Ang II). AT_1_R crystal structures either in an inactive or active state conformation provide detailed structural insights into this receptor (e.g., PDB IDs: 4zud [51], 6do1 [52], 6os0 [32]). To visualize our findings regarding loop mutants in the context of structural receptor properties, we used the (**A**) active state-like AT_1_R structure in complex with Ang II [32]. This active state AT_1_R structure (amino acids D9-Q315, PDB ID: 6os0) provided details of Ang II binding and extracellular loop (EL) conformations (different colours for EL1-3) and the N-terminal tail (Ntt). (**B**) Of note, beside the highly conserved G-protein coupled receptor (GPCR) disulfide bridge between a cysteine (yellow sticks) in transmembrane helix 3 (H3, C101) and a cysteine in the EL2 (C180), a second disulfide bridge fixes the N-terminus (C18) to the EL3/transmembrane helix 7 (H7) transition (C274). Amino acids studied here by blockwise alanine mutants are highlighted by line representation. Specific residues of the loops 1–3 are exposed to the extracellular solvent phase and may function as contact points for extracellularly interacting molecules.

To evaluate the potential impact of the ELs on the interaction of AT_1_R with AT_1_R-Abs in SRC, we designed and generated poly-alanine mutations in each of the three ELs and tested these mutants first on their capacity to express at the cell surface. Of note, two amino acid stretches in EL2 at positions 173-178 and 182-187 have been previously supposed to be epitopes involved in antibody-binding to AT_1_R [10,14]. Already determined AT_1_R structures revealed the spatial localization of these EL2 fragments, both being exposed to the extracellular solvent region accessible for ligand contacts (Figure 3A). Indeed, bound Ang II is in direct contact with the amino acid residues in the C-terminal EL2 fragment 182-187 (Figure 3B).

**Figure 4:**
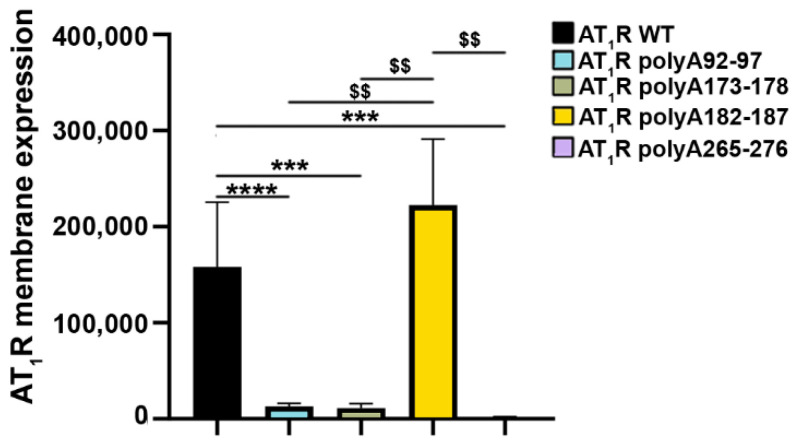
Angiotensin II type 1 receptor (AT_1_R) extracellular loop (EL)-variant cell-surface expression in mammalian cells. Human embryonic kidney 293T cells (HEK293Ts) were transfected with sequences coding for either HiBiT-tagged wild type (WT) receptors or HiBiT-tagged mutant receptors (amino acids 92 to 97, EL1 (AT_1_R polyA92-97); amino acids 173 to 178 and amino acids 182 to 187, EL2 (AT_1_R polyA173-178 and AT_1_R polyA182-187, respectively); and amino acids 265 to 276, EL3 (AT_1_R polyA265-276) to determine the cell-surface expression of the variants. SEMs derived from five to twelve experiments are shown together with *p*-values for Mann–Whitney tests (*** *p* < 0.001, **** *p* < 0.0001 as compared with AT_1_R WT without stimulation; $$ *p* < 0.01 as compared with AT_1_R polyA 182-187A).

All mutants were tagged with the HiBiT tag to evaluate how the alanine mutations affect the expression of AT_1_R at the cell surface. In HEK293T, we observed that most mutants, with the exception of AT_1_R polyA182-187, were significantly less expressed than AT_1_R WT (Figure 4). Variant AT_1_R polyA182-187 showed an expression level comparable to AT_1_R WT (Figure 4). Thus, only AT_1_R polyA182-187 was selected for further experiments.

**Figure 5:**
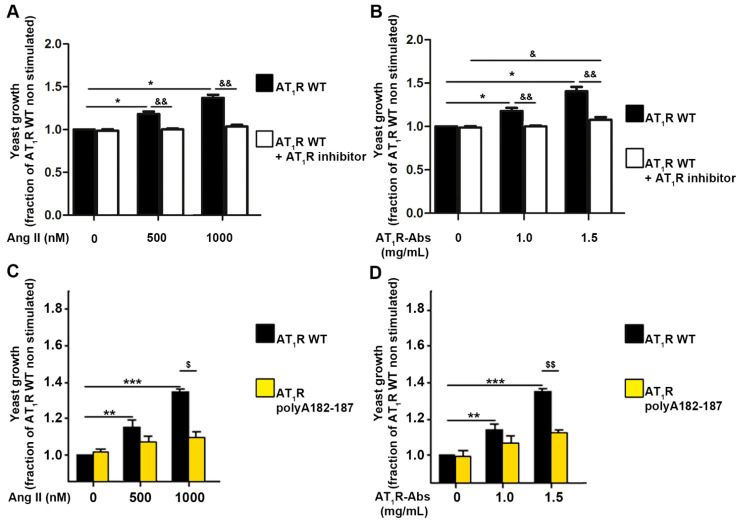
Contributions of extracellular loop 2 (EL2) to angiotensin II (Ang II) type 1 receptor (AT_1_R) activation. (**A**) Yeast growth is dose-dependently activated by Ang II. Yeasts transformed with a plasmid enabling the expression of the receptor were stimulated with increasing concentrations of Ang II, as indicated. Prior to stimulation, yeasts were incubated (or not) for one hour with the AT_1_R-inhibitor Valsartan at a concentration of 1 µM. (**B**) Human AT_1_R is dose-dependently activated by AT_1_R-Abs. Yeasts transformed with *AGTR1* were stimulated with increasing concentrations of antibodies. Prior to stimulation, yeasts were incubated or not for one hour with the AT_1_R-inhibitor Valsartan at a concentration of 1 µM. (**C**,**D**) Yeasts were transformed with the wild type receptor (AT_1_R WT) or with the receptor variant mutated at amino acids 182-187 (AT_1_R polyA182-187). Constructs were stimulated with increasing concentrations of Ang II (**C**) or AT_1_R-Abs (**D**). SEMs derived from four to six experiments are shown along with *p*-values for Wilcoxon or Mann–Whitney tests. (**A**,**B**) * *p* < 0.05 as compared with AT_1_R WT without stimulation and without inhibitorscompared with; & *p* < 0.05, && *p* < 0.01 as compared with AT_1_R WT pre-incubated with AT_1_R inhibitors (Valsartan). (**C**,**D**) ** *p* < 0.01, *** *p* < 0.001 as compared with AT_1_R WT without stimulation; $ *p* < 0.05, $$ *p* < 0.01 as compared with AT_1_R polyA182-187.)

As mentioned above, the patients whose material was investigated presented both AT_1_R-Abs and auto-antibodies targeting ET_A_Rs. To avoid cross-reactivity and obtain a direct read-out of the effects of structural changes on the receptor’s function, we opted to develop a MMY14-AT_1_R yeast system (described in Material and Methods). To first test the reliability of the yeast growth assay, we used human WT AT_1_R expressed in MMY14 yeast cells. Stimulation with Ang II revealed that human WT AT_1_R indeed responds dose-dependently to its endogenous peptide agonist, as indicated by increased cell growth (Figure 5A). In support, pre-incubation of yeast cells with the AT_1_R inhibitor (antagonist) Valsartan inhibited Ang II ligand-induced yeast growth, even at the highest concentration of supplemented Ang II (Figure 5A). Secondly, MMY14-AT_1_R stimulated with AT_1_R-Abs (isolated from patients with SRC) also induced positive yeast growth levels. AT_1_R-Abs dose-dependently activates AT_1_R, as shown by a growth level comparable to the level induced by Ang II (Figure 5B). Pre-incubation with Valsartan resulted in a statistically significant decrease in yeast growth compared with AT_1_R-Abs/AT_1_R-mediated cell growth. Altogether, these findings confirmed the reliability of the assay for testing AT_1_R-mediated signaling induced by ligands. Transformation of yeasts with alanine-mutant receptors bearing AT_1_R polyA182-187A revealed that Ang II and AT_1_R-Abs-mediated activation of the receptors was significantly impaired compared with yeasts transformed with the WT receptor (Figure 5C,D). This supposes an essential role of the EL2 in Abs-mediated signaling effects.

## 3. Discussion

### 3.1. AT_1_R Auto-Antibodies Inhibit Ang II-Mediated Receptor Internalization

We show here that AT_1_R-Abs action not only lacks subsequent receptor internalization but also attenuates Ang II-induced AT_1_R internalization or leads to a prolonged surface expression of the receptor (Figure 2). This effect is potentially associated with differences regarding β-arrestin recruitment and receptor phosphorylation, as supposed by a previous study [53].

By impairing β-arrestin recruitment, AT_1_R-Abs, in contrast to Ang II, are categorised as GPCR-biased ligands that only alter a specific signaling pathway compared with endogenous signaling [54]. This contrasts with most recent findings on AT_1_R-Abs in transplantation patients showing that these antibodies induced β2-arrestin recruitment [55]. However, previous studies have shown that β-arrestin signaling is privileged and has beneficial effects; for example, TRV120027, a β-arrestin biased ligand of AT_1_R, increases cardiomyocyte contraction and reduces Ang II effects in rats [56]. Similarly, TRV12023, another β-arrestin biased ligand of AT_1_R, increases cardiac contractility and induced pro-survival signaling after cardiac injury in mice [57]. Of note, TRV12023 also exhibited protective effects in a rat model infused with Ang II [58] by preventing cardiac hypertrophy of dilated cardiomyopathy [59]. Finally, β-arrestin-biased AT_1_R agonists have been suggested to treat COVID-19 in terms of inducing cardioprotective signaling pathways [36].

AT_1_R-Abs have been associated with increased intracellular signaling, particularly the ERK1/2 pathway, in SSc patients [26]. In vitro, AT_1_R-Abs induced higher IL-8 secretion by endothelial cells, resulting in decreased endothelial repair as well as increased monocyte migration and ROS production [60]. Moreover, these auto-antibodies triggered an increase in intracellular calcium in endothelial cells and higher vasoconstriction of rat pulmonary arteries ex vivo [61]. According to our findings, this stronger signaling might be due to a prolonged stay of the receptor at the cell surface and impaired signaling regulation.

In addition, a recently published study showed that heterodimerization of ET_A_R with endothelin-1 type B receptor (ET_B_R) could also delay β-arrestin recruitment, phosphorylation, and related receptor internalization [62]. Unfortunately, little is known yet regarding the heterodimerization capacity of endothelin with Ang II receptors. Therefore, we cannot exclude that AT_1_R heterodimerizes with ET_A_R in vivo, which may also have an impact on β-arrestin recruitment by receptors. Of note, antibodies targeting both receptors appear concomitantly in various diseases, such as preeclampsia, graft rejection, and systemic sclerosis [26,63,64,65].

Of note, we cannot exclude completely that the HiBiT tag affects AT_1_R internalization. However, recent data published by the Promega Company showed that the β2-adrenergic receptor tagged with HiBiT internalized in the same way and to the same extent as reported in previously published studies [66].

### 3.2. Cell Proliferation Is Mediated by G_q/11_ Activation and Inhibited by Basal G_i_ Signaling

G_q/11_ is activated by Ang II at the AT_1_R [67], also shown in tissues where Ang II triggers physiological effects [68]. Hence, G_q/11_ mediates the cardiac hypertrophic effects of Ang II involving vascular smooth muscle cells but not cardiomyocytes in transgenic mice [69], which was also confirmed ex vivo [7]. It has been demonstrated that neural progenitor proliferation was impaired by G_q/11_ inhibition [44]. Moreover, AT_1_R activation via Ang II results in the proliferation of Human Umbilical Vascular Endothelial Cells (HUVEC) and is associated with increased expression of Vascular Endothelial Growth Factor (VEGF) [70]. Recently, it was reported that G_q/11_ regulates VEGF-induced HUVEC proliferation, and inactivation of G_q/11_ resulted in vivo in neo-angiogenesis impairment [46]. These effects could be blocked with Candesartan, a specific AT_1_R inhibitor.

It should also be considered that AT_1_R heterodimerizes with different receptors, such as Bradykinin 2 receptors [71,72,73], these receptors constituting a potential secondary mechanism for G_q/11_ activation. Complementary experiments are required to prove such a mechanism.

Under our experimental in vitro conditions, the proliferating effect of Ang II on HMEC-1s was very limited and more pronounced for AT_1_R-Abs (Figure 1B). Although it is possible that the previously observed differences between the two stimuli resulted from the effects of AT_2_R with native HMEC-1s (which showed no proliferation with Ang II [25]), AT_2_R anti-proliferative effects (as described in vascular smooth muscle cells (VSMCs) [74]) are here very unlikely due to *AGTR1* mRNA being 100,000-fold more expressed than *AGTR2* mRNA (Supplementary Materials Figure S1). Furthermore, we recently showed that AT_2_R inhibitors fail to block SSc antibodies-induced endothelial cell proliferation [25]. In any case, further complementary studies are still required to determine the exact difference between Ang II- and antibodies-induced endothelial cell proliferation.

Our data also suggest that immune activation of AT_1_R by Abs stimulates G_q/11_-mediated signaling (Figure 1C) but not G_i_ signaling, which in turn increases endothelial cell proliferation. Notably, blocking basal Gi signaling activity strongly enhances cell proliferation induced by AT_1_R-Abs but not by Ang II (Figure 1D). This implies that, on the one hand, a basal G_i_ signaling activity may have a “protective” effect towards cell proliferation induced by Abs, but, on the other hand, it again indicates that Ang II and AT_1_R-Abs differ in their signaling effects. Interestingly, in 2016, it was reported that G_α/_i proteins are involved in the hyperproliferation of VSMCs in hypertensive rats [75], a finding confirmed in two other studies [76,77]. Similarly, another recent publication has shown that Gi-mediated signaling activity influences cancer cell proliferation [78]. However, why AT_1_R-Abs induces proliferation of HMEC-1 more efficiently than the natural ligand Ang II and how reduced basal G_i_ signaling enhances the proliferative effect of Abs needs to be addressed (Figure 1C).

The reason why AT_1_R-Abs induces a stronger proliferation of HMEC-1s than Ang II and how reduced basal G_i_ signaling contributes to an enhanced proliferative effect of Abs needs to be clarified in more detail in future work.

### 3.3. EL2 Contributes to Antibody-Mediated Signaling in Systemic Sclerosis

In addition to the auto-antibodies involved in preeclampsia, it has been suggested that AT_1_R-Abs found in transplant rejection recognize not just one epitope located in the AT_1_R EL2 but interact with at least two distinguishable epitopes in this loop [10,14]. In general, the EL2 among many GPCRs is known as one of the key elements in ligand binding and signaling regulation [79,80]. Our AT_1_Rs using blockwise alanine mutants also indicate that this receptor has an essential role in EL2 (Figure 5). In our experimental MMY model, an alanine mutation in the amino acid stretch 182 to 187 led to a complete inhibition of the receptor capability for signaling (Figure 5C,D), yet without specificity for immunogenic activation. We conclude that the amino acid composition and probably the associated spatial arrangement of the EL2 relative to other receptor components is important for antibody and simultaneously for ligand recognition (Figure 3) or signaling regulation. This is in accordance with structural information about the AT_1_R-Ang II complex that shows that Ang II directly interacts with EL2 amino acids, further confirmed by binding studies of Ang II at the AT_1_R [30]. An additional contribution of Abs binding at particular EL2 parts must be assumed. Altogether, modifications at this loop, like the alanine substitutions tested here, disturb the ligand–peptide binding pocket and/or modify antibody-recognition motifs (Figure 3).

To date, only limited information is available on the potential contribution of AT_1_R EL1 and EL3 for ligand and Abs binding. Both loops expose various amino acids to the extracellular site, which may function as antibody epitopes (Figure 3). Between 1995 and 2003, studies were published about peptides derived from the first EL of rat AT_1_R which aimed to determine the structure of this loop [81]. However, no functional characterization accompanied these approaches. Our alanine substitutions of EL1 and EL3 led to impaired cell-surface expression (Figure 4), at least in HEK293T cells, which makes it unfortunately impossible to exclude or infer their contribution to AT_1_R-Abs binding.

### 3.4. Conclusions

Altogether, our investigations revealed that Ang II-induced internalization of AT_1_R is attenuated by a simultaneous action of AT_1_R-Abs. Importantly, G_q/11_ but not G_i_ activation induced by AT_1_R-Abs mediates cell proliferation. Furthermore, G_i_ basal signaling activity of AT_1_R has a decreasing effect on cell proliferation; inhibition of G_i_ basal activity potentiates the proliferation-enhancing effect of AT_1_R-Abs but not Ang II, which requires further clarification of the molecular causes. In summary, our data show that Ang II and AT_1_R-Abs elicit different effects in terms of cell proliferation and G_q/11_ activation in patients with SRC, at least under our experimental conditions. This current study, therefore, provides insights into the molecular action of AT_1_R-Abs and the associated mechanisms of related pathogenesis.

## 4. Materials and Methods

### 4.1. Clinical Samples of Patients with Systemic Sclerosis

Plasma was obtained from three patients treated for Angiotensin Converting Enzyme I (ACEI) refractory SRC in our clinic (Charité—Universitätsmedizin Berlin, Germany) between January 2006 and October 2010. SRC was defined in terms of an otherwise unexplained rapid decline in renal function (increase in serum creatinine ≥ 50%) in a patient with systemic sclerosis. Diagnosis was confirmed by renal biopsy that assessed obliterative vasculopathy of arteries and arterioles in all cases. Moreover, all SRC individuals were tested for the presence of AT_1_R antibodies, as previously described (CellTrend GmbH, Luckenwalde, Germany) [25,26,61]. Patients were considered positive if their antibody level reached at least 17 U/mL [26]. The local Medical Ethics Committee approved the study protocol (EA1/013/705). All experiments were conducted with immunoglobulin G (IgG) extracted from patient plasma samples (AT_1_R-Abs), as previously described [14,25,26]. Based on the results obtained with the MMY yeast model in which AT_1_R was the sole GPCR expressed (see the description of the experimental model below), where AT_1_R-Abs dose-dependently activated AT_1_Rs (Figure 5B), we decided to perform the experiments with the smallest dose of 1 mg/mL activating AT_1_R.

### 4.2. Expression of Constructs and Site-Directed Mutagenesis

Complementary DNA of human full-length AT_1_R (kindly given by the group of Pierre Lavigne, Université de Sherbrook, Sherbrook, QC, Canada) was cloned into p426GPD (kindly given by Simon Dowell, GSK, Stevenage, United Kingdom) for expression into *S. cerevisiae*. Sub-cloning into pcDNA3 was also performed to allow expression in Human Microvascular Endothelial-1 cells (HMEC-1s) using the InFusion kit from Takara/Clontech (Mountain View, CA, USA). Site-directed mutagenesis was performed with the Q5 Site-directed mutagenesis kit from New England Biolabs (Frankfurt am Main, Germany) using specific primers (Table 1).

### 4.3. Yeast Culture and Transformation

p426GPD plasmids carrying sequences for wild type (WT) or mutated human AT_1_R were transformed into *S. cerevisiae* cultured in yeast extract peptone dextrose medium (YPD medium) according to the LiAc/SS carrier DNA/PEG procedure. The strain is derived from the MMY14 yeast strain [82] and was modified to communicate with mammalian GPCRs via the insertion of a chimeric G-protein [83] corresponding to G_q_ in this case. The full genotype of the strain is W303-1A *fus1::FUS1-HIS3 FUS1-lacZ::LEU2 far1_::ura3_gpa1_::ADE2_ sst2_::ura3 ste2_::G418^R^ TRP1*::*Gpa1/G_α_^q(5)^* [84].

### 4.4. Cell Culture and Transfection

HMEC-1s were cultured in MCDB 131 medium (cc pro GmbH, Oberdorla, Germany) supplemented with 5% fetal calf serum (FCS) (Invitrogen or Gibco, Waltham, MA, USA), glutamine, penicillin-streptomycin, human EGF, and hydrocortisone (complete medium). HMEC-1s were transfected using XFect (Takara, Mountain View, CA, USA) in MCDB 131 Medium supplemented with 0.5% FCS (starvation medium), according to the manufacturer’s instructions. Flasks and wells were coated with 0.2% gelatine.

HEK293T cells were cultured in DMEM High Glucose supplemented with 10% FCS, glutamine, penicillin-streptomycin, 4-(2-hydroxyethyl)-1-piperazineethanesulfonic acid (HEPES), and sodium pyruvate (complete medium). HEK293T cells were transfected using Lipofectamine 3000 (Invitrogen, Waltham, MA, USA) in DMEM high glucose without FCS (starvation medium), according to the manufacturer’s instructions. Wells were coated with Poly-l-lysine (Sigma Aldrich, Saint Louis, MI, USA). Cultures were maintained at 37 °C under 5% CO_2_.

For stimulation, HMEC-1 and HEK293T cells were cultured in starvation medium.

### 4.5. GPCR-Induced Yeast-Growth Assay

A specific yeast growth assay enabled a controlled activation and characterization of a particular GPCR. The methods and protocols used in this assay are well-established [84,85]. In brief, modified yeast strains grow under dependency of activation by a single inserted human GPCR. Furthermore, these yeasts express a chimeric yeast G-protein with five amino acids of a particular human G-protein C-terminus, conferring to each strain a forced human G-protein specificity. Upon activation of the transformed human GPCR, the chimeric G-protein is activated and triggers the activation of the Mitogen Activated Protein Kinase (MAPK) signaling pathway, resulting in the pairing and growing of the yeasts. This system has in principle already been demonstrated by studies with human adenosine 1 and 2B receptor subtypes [85,86]. Here, in analogy, MMY14 *S. cerevisiae* yeast were transformed with human AT_1_R (MMY14-AT_1_R) and, upon activation of the receptor, G_q/11_ activation could induce yeast growth. This assay, finally, allows detection of constitutively activated receptors and/or receptors with increased response to external stimuli through increased growth rate levels.

The transformed yeasts grew for three days on WHAUL plates supplemented in histidine (WHAUL-His). Four clones were picked-up and transferred individually onto a new WHAUL-His plate. On the day after, each clone was transferred into liquid WHAUL-His medium and allowed to grow overnight at 30 °C under shaking. To evaluate the activation of the receptor in response to stimulation, each pre-cultured clone was added to liquid WHAUL Medium without histidine and supplemented with BU salts, fluorescein di-β-d-galactopyranoside (10 nM final) to monitor yeast growth, and 3-amino-1,2,4 triazole (2 mM final) to suppress basal growth in His-deficient medium.

First, this system was stimulated with increasing concentrations of Ang II ranging from 0 to 1 µM to prove the reliability of the system for experimental studies, then we investigated the ligand-mediated action of WT AT_1_Rs or AT_1_R variants on yeast growth. AT_1_R-Abs were isolated as described above and dialyzed against WHAUL medium. Yeast growth was measured on a FLUOStar OPTIMA reader after 20 h incubation.

### 4.6. Luciferase Reporter Assay

HMEC-1s were plated on 24-well plates with 50,000 cells per well and allowed to grow in complete medium until they reached 70% confluence. Cells were then transfected with 250 ng WT or mutated human AT_1_R cloned into pcDNA3 and 250 ng NFAT luciferase reporter plasmids. Medium was changed 24 h after to complete medium. On the next day, the medium was changed to starvation medium with or without 5 ng/mL pertussis toxin. After one hour, cells were treated with medium with or without AT_1_R-Abs (1 mg/mL). AT_1_R-Abs were previously dialyzed against Dulbecco’s Modified Eagle’s Medium low Glucose for six hours. The cells were subsequently washed in PBS and lysed in 1x passive lysis buffer (Promega, Madison, WI, USA) according to the manufacturer’s instructions. Luciferase production was measured (luciferase assay system, Promega Corporation, Madison, WI, USA) according to the manufacturer’s instructions.

### 4.7. EdU Proliferation Assay

HMEC-1s were plated and cultivated as described above for the luciferase reporter assays. Transient transfection was performed with 250 ng of wild type or mutated human AT_1_Rs cloned into pcDNA3. The next day, the medium was changed to complete medium. After 24 h, the medium was changed to starvation medium with or without 5 ng/mL pertussis toxin. After one hour, cells were stimulated with medium with or without 1 mg/mL AT_1_R-Abs (dialyzed against DMEM Low Glucose for 24 h). Two hours prior to the end of stimulation, half the medium was replaced with starvation medium containing 20 µM edU. Immunofluorescence was performed according to the manufacturer’s instructions (iclick EdU Andy Fluor 488 Imaging kit, ABP Biosciences, Beltsville, MD, USA) and nuclei were counter-labelled with a PBS/DAPI 5 µg/mL solution.

### 4.8. Receptor Expression at the Cell Surface

HEK293T or HMEC-1 cells were plated on white 96-well plates (Corning, NY, USA) with 25,000 or 50,000 cells per well, respectively, and allowed to grow in complete medium. Cells were then transfected with 400 ng human AT_1_Rs tagged with HiBiT cloned into pcDNA3. Four hours after transfection, the medium was changed to complete medium. The cells were then incubated in starvation medium for a further day. On the next day, the cells were treated with the Nano-Glo HiBiT extracellular detection system (Promega Corporation, Madison, WI, USA) according to the manufacturer’s instructions.

### 4.9. Receptor Internalization after Ligand Exposure

HEK293T cells were plated on white 96-well plates (Corning, NY, USA) with 25,000 cells per well and allowed to grow in complete medium. Cells were then transfected with 400 ng human AT_1_Rs tagged with HiBiT cloned into pcDNA3. Four hours after transfection, the medium was changed to complete medium. The cells were then incubated in starvation medium for a further day. On the day of stimulation, HEK293T cells were incubated for different durations (10 min, 3 and 6 h) and stimulated with 1 µM Ang II or 1 mg/mL AT_1_R-Abs (dialyzed against DMEM Low Glucose for 24 h).

Alternatively, HEK293T cells were pre-incubated with AT_1_R-Abs (1 mg/mL) or DMEM low glucose for six hours, then stimulated with different concentrations of Ang II (10 nM, 100 nM, or 1 µM) for 10 min.

In every case, cells were treated following stimulation with the Nano-Glo HiBiT extracellular detection system (Promega Corporation, Madison, WI, USA) according to the manufacturer’s instructions.

### 4.10. Statistics

All experiments were performed with individual antibodies from two to three patients, each experiment being repeated five to six times. GraphPad Prism v5.00 software (GraphPad software, San Diego, CA, USA) was used to perform all statistical analyses. Wilcoxon and Mann–Whitney U tests were conducted as appropriate (*, #, § *p* < 0.05, **, ##, §§ *p* < 0.01, *** *p* < 0.001). Data are all presented as means ± SEM.

## Figures and Tables

**Table 1:** Primer sequences for site-directed mutagenesis.

Mutation	Forward Primer (5′ to 3′)	Reverse Primer (5′ to 3′)
polyA265-276	AGCTGCCGCTGCAGCTGCA GATATTGTGGACACGGCC	GCGGCGGCTGCTGCAGCCGC TACATCCAGAAAAGTGAATATTTG
polyA173-178	GCTGCTGCCGTTTGTGCTTTCCATTATGAG	GGCGGCCGCAATGAAAAATACATTTCGATGGATTATAG
polyA182-187	TGCGGCCGCAAATTCAACCCTTCCGATAG	GCAGCGGCAGCACAAACTGTAATATTGGTGTTC
polyA92-97	GCCGCTGCCAATTACCTATGTAAGATTGCTTC	CGCGGCGGCTTCCATAGCTGTGTAGAC

## Data Availability

The data presented in this study are available on request from the corresponding authors. The data are not publicly available as they may be used for further studies by the group.

## Supplementary Materials

The following supporting information can be downloaded at: https://www.mdpi.com/article/10.3390/ijms23073984/s1.

## Abbreviations

ACEI	angiotensin converting enzyme I
ACEi	angiotensin converting enzyme inhibitors
Ang II	angiotensin II
AP1	Activator Protein 1
ARB	Angiotensin Receptor Blockers
AT_1_R	angiotensin II type 1 receptor
AT_2_R	angiotensin II type 2 receptor
AT1R-Abs	angiotensin II type 1 receptor auto-antibodies
EL	extracellular loop
ERK1/2	extracellular signal-regulated kinases 1/2
ET_A_R	endothelin-1 type A receptor
ET_B_R	endothelin-1 type B receptor
FCS	fetal calf serum
GPCR	G-protein coupled receptor
HEK293T	human embryonic kidney 293T cells
HMEC-1	human microvascular endothelial cells
HEPES	4-(2-hydroxyethyl)-1-piperazineethanesulfonic acid
HUVEC	human umbilical vascular endothelial cells
IgG	immunoglobulin G
MAPK	mitogen activated protein kinase
NFAT	nuclear factor of activated T-cells
NFκB	Nuclear Factor of “κappa-light-chain-enhancer” of activated B-cells
Ntt	N-terminal tail
PTx	pertussis toxin
RAAS	renin-angiotensin-aldosterone system
SARS-CoV-2	severe acute respiratory syndrome coronavirus-2
SSc	systemic sclerosis
SRC	scleroderma renal crisis
TM	transmembrane helix
TSHR	thyroid stimulating hormone receptor
VSMC	vascular smooth muscle cells
VEGF	vascular endothelial growth factor
WT	wild-type

## References

[1] Fredriksson R., Lagerstrom M.C., Lundin L.G., Schioth H.B. (2003). The G-protein-coupled receptors in the human genome form five main families. Phylogenetic analysis, paralogon groups, and fingerprints. Mol. Pharmacol..

[2] Hofmann K.P., Scheerer P., Hildebrand P.W., Choe H.W., Park J.H., Heck M., Ernst O.P. (2009). A G protein-coupled receptor at work: The rhodopsin model. Trends Biochem. Sci..

[3] Allen A.M., Zhuo J., Mendelsohn F.A. (1999). Localization of angiotensin AT1 and AT2 receptors. J. Am. Soc. Nephrol..

[4] Abe K., Nakashima H., Ishida M., Miho N., Sawano M., Soe N.N., Kurabayashi M., Chayama K., Yoshizumi M., Ishida T. (2008). Angiotensin II-induced osteopontin expression in vascular smooth muscle cells involves Gq/11, Ras, ERK, Src and Ets-1. Hypertens Res..

[5] Hunyady L., Catt K.J. (2006). Pleiotropic AT1 receptor signaling pathways mediating physiological and pathogenic actions of angiotensin II. Mol. Endocrinol..

[6] Miserey-Lenkei S., Lenkei Z., Parnot C., Corvol P., Clauser E. (2001). A functional enhanced green fluorescent protein (EGFP)-tagged angiotensin II at(1a) receptor recruits the endogenous Galphaq/11 protein to the membrane and induces its specific internalization independently of receptor-g protein coupling in HEK-293 cells. Mol. Endocrinol..

[7] Ohtsu H., Higuchi S., Shirai H., Eguchi K., Suzuki H., Hinoki A., Brailoiu E., Eckhart A.D., Frank G.D., Eguchi S. (2008). Central role of Gq in the hypertrophic signal transduction of angiotensin II in vascular smooth muscle cells. Endocrinology.

[8] Laragh J.H., Angers M., Kelly W.G., Lieberman S. (1960). Hypotensive agents and pressor substances. The effect of epinephrine, norepinephrine, angiotensin II, and others on the secretory rate of aldosterone in man. JAMA.

[9] Mouw D., Bonjour J.P., Malvin R.L., Vander A. (1971). Central action of angiotensin in stimulating ADH release. Am. J. Physiol..

[10] Wallukat G., Homuth V., Fischer T., Lindschau C., Horstkamp B., Jupner A., Baur E., Nissen E., Vetter K., Neichel D. (1999). Patients with preeclampsia develop agonistic autoantibodies against the angiotensin AT1 receptor. J. Clin. Invest..

[11] Lefaucheur C., Viglietti D., Bouatou Y., Philippe A., Pievani D., Aubert O., Duong Van Huyen J.P., Taupin J.L., Glotz D., Legendre C. (2019). Non-HLA agonistic anti-angiotensin II type 1 receptor antibodies induce a distinctive phenotype of antibody-mediated rejection in kidney transplant recipients. Kidney. Int..

[12] Sas-Strozik A., Donizy P., Koscielska-Kasprzak K., Kaminska D., Gawlik K., Mazanowska O., Madziarska K., Halon A., Krajewska M., Banasik M. (2020). Angiotensin II type 1 receptor expression in renal transplant biopsies and anti-AT1R antibodies in serum indicates the risk of transplant loss. Transplant. Proc..

[13] Wozniak L.J., Hickey M.J., Chan A.P., Venick R.S., Farmer D.G., Busuttil R.W., Reed E.F., McDiarmid S.V. (2020). Angiotensin II type-1 receptor antibodies are associated with active allograft dysfunction following pediatric liver transplantation. Transplantation.

[14] Dragun D., Muller D.N., Brasen J.H., Fritsche L., Nieminen-Kelha M., Dechend R., Kintscher U., Rudolph B., Hoebeke J., Eckert D. (2005). Angiotensin II type 1-receptor activating antibodies in renal-allograft rejection. N. Engl. J. Med..

[15] Hinchcliff M., Varga J. (2007). Obliterative vasculopathy in systemic sclerosis: Endothelial precursor cells as novel targets for therapy. Expert. Rev. Clin. Immunol..

[16] Kuwana M., Kaburaki J., Okazaki Y., Yasuoka H., Kawakami Y., Ikeda Y. (2006). Increase in circulating endothelial precursors by atorvastatin in patients with systemic sclerosis. Arthritis. Rheum..

[17] Dragun D., Catar R., Philippe A. (2016). Non-HLA antibodies against endothelial targets bridging allo- and autoimmunity. Kidney Int..

[18] Sas-Strozik A., Krajewska M., Banasik M. (2020). The significance of angiotensin II type 1 receptor (AT1 receptor) in renal transplant injury. Adv. Clin. Exp. Med..

[19] Sorohan B.M., Ismail G., Leca N., Tacu D., Obrisca B., Constantinescu I., Baston C., Sinescu I. (2020). Angiotensin II type 1 receptor antibodies in kidney transplantation: An evidence-based comprehensive review. Transplant. Rev..

[20] Zhang X., Reinsmoen N.L. (2019). Angiotensin II type I receptor antibodies in thoracic transplantation. Hum. Immunol..

[21] Zhang X., Reinsmoen N.L. (2020). Impact and production of Non-HLA-specific antibodies in solid organ transplantation. Int. J. Immunogenet..

[22] Liu F., Wang Y.X., Wang X.F., Zheng Y.Q., Jin Z., Zhi J.M. (2016). Role of agonistic autoantibodies against type-1 angiotensin II receptor in the pathogenesis of retinopathy in preeclampsia. Sci. Rep..

[23] Siddiqui A.H., Irani R.A., Zhang W., Wang W., Blackwell S.C., Kellems R.E., Xia Y. (2013). Angiotensin receptor agonistic autoantibody-mediated soluble fms-like tyrosine kinase-1 induction contributes to impaired adrenal vasculature and decreased aldosterone production in preeclampsia. Hypertension.

[24] Zhou C.C., Ahmad S., Mi T., Abbasi S., Xia L., Day M.C., Ramin S.M., Ahmed A., Kellems R.E., Xia Y. (2008). Autoantibody from women with preeclampsia induces soluble Fms-like tyrosine kinase-1 production via angiotensin type 1 receptor and calcineurin/nuclear factor of activated T-cells signaling. Hypertension.

[25] Catar R., Herse-Naether M., Zhu N., Wagner P., Wischnewski O., Kusch A., Kamhieh-Milz J., Eisenreich A., Rauch U., Hegner B. (2021). Autoantibodies targeting AT1- and ETA-receptors link endothelial proliferation and coagulation via Ets-1 transcription factor. Int. J. Mol. Sci..

[26] Riemekasten G., Philippe A., Nather M., Slowinski T., Muller D.N., Heidecke H., Matucci-Cerinic M., Czirjak L., Lukitsch I., Becker M. (2011). Involvement of functional autoantibodies against vascular receptors in systemic sclerosis. Ann. Rheum. Dis..

[27] Clement M.J., Fortune A., Phalipon A., Marcel-Peyre V., Simenel C., Imberty A., Delepierre M., Mulard L.A. (2006). Toward a better understanding of the basis of the molecular mimicry of polysaccharide antigens by peptides: The example of Shigella flexneri 5a. J. Biol. Chem..

[28] Fillion D., Cabana J., Guillemette G., Leduc R., Lavigne P., Escher E. (2013). Structure of the human angiotensin II type 1 (AT1) receptor bound to angiotensin II from multiple chemoselective photoprobe contacts reveals a unique peptide binding mode. J. Biol. Chem..

[29] Laporte S.A., Boucard A.A., Servant G., Guillemette G., Leduc R., Escher E. (1999). Determination of peptide contact points in the human angiotensin II type I receptor (AT1) with photosensitive analogs of angiotensin II. Mol. Endocrinol..

[30] Unal H., Jagannathan R., Bhat M.B., Karnik S.S. (2010). Ligand-specific conformation of extracellular loop-2 in the angiotensin II type 1 receptor. J. Biol. Chem..

[31] Unal H., Jagannathan R., Bhatnagar A., Tirupula K., Desnoyer R., Karnik S.S. (2013). Long range effect of mutations on specific conformational changes in the extracellular loop 2 of angiotensin II type 1 receptor. J. Biol. Chem..

[32] Wingler L.M., Skiba M.A., McMahon C., Staus D.P., Kleinhenz A.L.W., Suomivuori C.M., Latorraca N.R., Dror R.O., Lefkowitz R.J., Kruse A.C. (2020). Angiotensin and biased analogs induce structurally distinct active conformations within a GPCR. Science.

[33] Trougakos I.P., Stamatelopoulos K., Terpos E., Tsitsilonis O.E., Aivalioti E., Paraskevis D., Kastritis E., Pavlakis G.N., Dimopoulos M.A. (2021). Insights to SARS-CoV-2 life cycle, pathophysiology, and rationalized treatments that target COVID-19 clinical complications. J. Biomed. Sci..

[34] Bellis A., Mauro C., Barbato E., Trimarco B., Morisco C. (2020). The rationale for angiotensin receptor neprilysin inhibitors in a multi-targeted therapeutic approach to COVID-19. Int. J. Mol. Sci..

[35] Sharma T., Mehan S. (2020). Possible therapeutic interventions in COVID-19 induced ARDS by cotinine as an ACE-2 promoter and AT-1R blocker. Infect. Disord. Drug Targets..

[36] Manglik A., Wingler L.M., Rockman H.A., Lefkowitz R.J. (2020). Beta-arrestin-biased angiotensin II receptor agonists for COVID-19. Circulation.

[37] Kowal-Bielecka O., Fransen J., Avouac J., Becker M., Kulak A., Allanore Y., Distler O., Clements P., Cutolo M., Czirjak L. (2017). Update of EULAR recommendations for the treatment of systemic sclerosis. Ann. Rheum. Dis..

[38] Nagaraja V. (2019). Management of scleroderma renal crisis. Curr. Opin. Rheumatol..

[39] Bruni C., Cometi L., Gigante A., Rosato E., Matucci-Cerinic M. (2021). Prediction and primary prevention of major vascular complications in systemic sclerosis. Eur. J. Intern. Med..

[40] Gordon S.M., Hughes J.B., Nee R., Stitt R.S., Bailey W.T., Little D.J., Edison J.D., Olson S.W. (2019). Systemic sclerosis medications and risk of scleroderma renal crisis. BMC Nephrol..

[41] Butikofer L., Varisco P.A., Distler O., Kowal-Bielecka O., Allanore Y., Riemekasten G., Villiger P.M., Adler S., Collaborators E. (2020). ACE inhibitors in SSc patients display a risk factor for scleroderma renal crisis-a EUSTAR analysis. Arthritis. Res. Ther..

[42] Gomez Sandoval Y.H., Levesque L.O., Anand-Srivastava M.B. (2009). Contribution of epidermal growth factor receptor transactivation in angiotensin II-induced enhanced expression of Gi protein and proliferation in A10 vascular smooth muscle cells. Can. J. Physiol. Pharmacol..

[43] Boss V., Talpade D.J., Murphy T.J. (1996). Induction of NFAT-mediated transcription by Gq-coupled receptors in lymphoid and non-lymphoid cells. J. Biol. Chem..

[44] Morishita R., Ueda H., Ito H., Takasaki J., Nagata K., Asano T. (2007). Involvement of Gq/11 in both integrin signal-dependent and -independent pathways regulating endothelin-induced neural progenitor proliferation. Neurosci. Res..

[45] Sarkar O., Li Y., Anand-Srivastava M.B. (2017). Nitric oxide attenuates overexpression of Gialpha proteins in vascular smooth muscle cells from SHR: Role of ROS and ROS-mediated signaling. PLoS ONE.

[46] Sivaraj K.K., Li R., Albarran-Juarez J., Wang S., Tischner D., Grimm M., Swiercz J.M., Offermanns S., Wettschureck N. (2015). Endothelial Galphaq/11 is required for VEGF-induced vascular permeability and angiogenesis. Cardiovasc. Res..

[47] Hsia J.A., Moss J., Hewlett E.L., Vaughan M. (1984). ADP-ribosylation of adenylate cyclase by pertussis toxin. Effects on inhibitory agonist binding. J. Biol. Chem..

[48] Raymond J.R. (1995). Multiple mechanisms of receptor-G protein signaling specificity. Am. J. Physiol..

[49] Thomas W.G., Thekkumkara T.J., Baker K.M. (1996). Molecular mechanisms of angiotensin II (AT1a) receptor endocytosis. Clin. Exp. Pharmacol. Physiol..

[50] Bian J., Lei J., Yin X., Wang P., Wu Y., Yang X., Wang L., Zhang S., Liu H., Fu M.L.X. (2019). Limited AT1 receptor internalization is a novel mechanism underlying sustained vasoconstriction induced by AT1 receptor autoantibody from preeclampsia. J. Am. Heart Assoc..

[51] Zhang H., Unal H., Gati C., Han G.W., Liu W., Zatsepin N.A., James D., Wang D., Nelson G., Weierstall U. (2015). Structure of the Angiotensin receptor revealed by serial femtosecond crystallography. Cell.

[52] Wingler L.M., McMahon C., Staus D.P., Lefkowitz R.J., Kruse A.C. (2019). Distinctive activation mechanism for angiotensin receptor revealed by a synthetic nanobody. Cell.

[53] Qian H., Pipolo L., Thomas W.G. (2001). Association of beta-Arrestin 1 with the type 1A angiotensin II receptor involves phosphorylation of the receptor carboxyl terminus and correlates with receptor internalization. Mol. Endocrinol..

[54] Mohammad Nezhady M.A., Rivera J.C., Chemtob S. (2020). Location bias as emerging paradigm in GPCR biology and drug discovery. iScience.

[55] Catar R.A., Wischnewski O., Chen L., Heidecke H., Rutz C., Schulein R., Dragun D., Philippe A., Kusch A. (2021). Non-HLA antibodies targeting angiotensin II type 1 receptors and endothelin-1 type A receptors impair endothelial repair via a beta2-arrestin link to the mTOR pathway. Kidney Int..

[56] Violin J.D., DeWire S.M., Yamashita D., Rominger D.H., Nguyen L., Schiller K., Whalen E.J., Gowen M., Lark M.W. (2010). Selectively engaging beta-arrestins at the angiotensin II type 1 receptor reduces blood pressure and increases cardiac performance. J. Pharmacol. Exp. Ther..

[57] Kim K.S., Abraham D., Williams B., Violin J.D., Mao L., Rockman H.A. (2012). beta-Arrestin-biased AT1R stimulation promotes cell survival during acute cardiac injury. Am. J. Physiol. Heart Circ. Physiol..

[58] Monasky M.M., Taglieri D.M., Henze M., Warren C.M., Utter M.S., Soergel D.G., Violin J.D., Solaro R.J. (2013). The beta-arrestin-biased ligand TRV120023 inhibits angiotensin II-induced cardiac hypertrophy while preserving enhanced myofilament response to calcium. Am. J. Physiol. Heart Circ. Physiol..

[59] Tarigopula M., Davis R.T., Mungai P.T., Ryba D.M., Wieczorek D.F., Cowan C.L., Violin J.D., Wolska B.M., Solaro R.J. (2015). Cardiac myosin light chain phosphorylation and inotropic effects of a biased ligand, TRV120023, in a dilated cardiomyopathy model. Cardiovasc. Res..

[60] Kill A., Tabeling C., Undeutsch R., Kuhl A.A., Gunther J., Radic M., Becker M.O., Heidecke H., Worm M., Witzenrath M. (2014). Autoantibodies to angiotensin and endothelin receptors in systemic sclerosis induce cellular and systemic events associated with disease pathogenesis. Arthritis. Res. Ther..

[61] Becker M.O., Kill A., Kutsche M., Guenther J., Rose A., Tabeling C., Witzenrath M., Kuhl A.A., Heidecke H., Ghofrani H.A. (2014). Vascular receptor autoantibodies in pulmonary arterial hypertension associated with systemic sclerosis. Am. J. Respir. Crit. Care Med..

[62] Zrein A., Bagher A.M., Young A.P., Denovan-Wright E.M., Kelly M.E.M. (2020). Endothelin receptor heteromerization inhibits beta-arrestin function in HEK293 cells. Can. J. Physiol. Pharmacol..

[63] Banasik M., Boratynska M., Koscielska-Kasprzak K., Kaminska D., Zmonarski S., Mazanowska O., Krajewska M., Bartoszek D., Zabinska M., Myszka M. (2014). Non-HLA antibodies: Angiotensin II type 1 receptor (anti-AT1R) and endothelin-1 type A receptor (anti-ETAR) are associated with renal allograft injury and graft loss. Transplant. Proc..

[64] Buttrup Larsen S., Wallukat G., Schimke I., Sandager A., Tvilum Christensen T., Uldbjerg N., Torring N. (2018). Functional autoantibodies against Endothelin-1 receptor type A and Angiotensin II receptor type 1 in patients with preeclampsia. Pregnancy Hypertens.

[65] Hiemann N.E., Meyer R., Wellnhofer E., Schoenemann C., Heidecke H., Lachmann N., Hetzer R., Dragun D. (2012). Non-HLA antibodies targeting vascular receptors enhance alloimmune response and microvasculopathy after heart transplantation. Transplantation.

[66] Boursier M.E., Levin S., Zimmerman K., Machleidt T., Hurst R., Butler B.L., Eggers C.T., Kirkland T.A., Wood K.V., Friedman Ohana R. (2020). The luminescent HiBiT peptide enables selective quantitation of G protein-coupled receptor ligand engagement and internalization in living cells. J. Biol. Chem..

[67] Murphy T.J., Alexander R.W., Griendling K.K., Runge M.S., Bernstein K.E. (1991). Isolation of a cDNA encoding the vascular type-1 angiotensin II receptor. Nature.

[68] de Gasparo M., Catt K.J., Inagami T., Wright J.W., Unger T. (2000). International union of pharmacology. XXIII. The angiotensin II receptors. Pharmacol. Rev..

[69] Keys J.R., Greene E.A., Koch W.J., Eckhart A.D. (2002). Gq-coupled receptor agonists mediate cardiac hypertrophy via the vasculature. Hypertension.

[70] Herr D., Rodewald M., Fraser H.M., Hack G., Konrad R., Kreienberg R., Wulff C. (2008). Regulation of endothelial proliferation by the renin-angiotensin system in human umbilical vein endothelial cells. Reproduction.

[71] AbdAlla S., Lother H., Quitterer U. (2000). AT1-receptor heterodimers show enhanced G-protein activation and altered receptor sequestration. Nature.

[72] Anton E.L., Fernandes D., Assreuy J., da Silva-Santos J.E. (2019). Bradykinin increases BP in endotoxemic rat: Functional and biochemical evidence of angiotensin II AT1 /bradykinin B2 receptor heterodimerization. Br. J. Pharmacol..

[73] Wilson P.C., Lee M.H., Appleton K.M., El-Shewy H.M., Morinelli T.A., Peterson Y.K., Luttrell L.M., Jaffa A.A. (2013). The arrestin-selective angiotensin AT1 receptor agonist [Sar1,Ile4,Ile8]-AngII negatively regulates bradykinin B2 receptor signaling via AT1-B2 receptor heterodimers. J. Biol. Chem..

[74] Nakajima M., Hutchinson H.G., Fujinaga M., Hayashida W., Morishita R., Zhang L., Horiuchi M., Pratt R.E., Dzau V.J. (1995). The angiotensin II type 2 (AT2) receptor antagonizes the growth effects of the AT1 receptor: Gain-of-function study using gene transfer. Proc. Natl. Acad. Sci. USA.

[75] Bou Daou G., Li Y., Anand-Srivastava M.B. (2016). Enhanced expression of Gialpha proteins contributes to the hyperproliferation of vascular smooth muscle cells from spontaneously hypertensive rats via MAP kinase- and PI3 kinase-independent pathways. Can. J. Physiol. Pharmacol..

[76] Hossain E., Li Y., Anand-Srivastava M.B. (2021). Angiotensin II-induced overexpression of sirtuin 1 contributes to enhanced expression of Gialpha proteins and hyperproliferation of vascular smooth muscle cells. Am. J. Physiol. Heart Circ. Physiol..

[77] Li Y., Hossain E., Arifen N., Srivastava A.K., Anand-Srivastava M.B. (2022). Sirtuin1 contributes to the overexpression of Gialpha proteins and hyperproliferation of vascular smooth muscle cells from spontaneously hypertensive rats. J. Hypertens.

[78] Lyu C., Ye Y., Lensing M.M., Wagner K.U., Weigel R.J., Chen S. (2021). Targeting Gi/o protein-coupled receptor signaling blocks HER2-induced breast cancer development and enhances HER2-targeted therapy. JCI Insight.

[79] Massotte D., Kieffer B.L. (2005). The second extracellular loop: A damper for G protein-coupled receptors?. Nat. Struct. Mol. Biol..

[80] Peeters M.C., van Westen G.J., Li Q., AP I.J. (2011). Importance of the extracellular loops in G protein-coupled receptors for ligand recognition and receptor activation. Trends Pharmacol. Sci..

[81] Nicastro G., Peri F., Franzoni L., de Chiara C., Sartor G., Spisni A. (2003). Conformational features of a synthetic model of the first extracellular loop of the angiotensin II AT1A receptor. J. Pept. Sci..

[82] Olesnicky N.S., Brown A.J., Dowell S.J., Casselton L.A. (1999). A constitutively active G-protein-coupled receptor causes mating self-compatibility in the mushroom Coprinus. EMBO J..

[83] Brown A.J., Dyos S.L., Whiteway M.S., White J.H., Watson M.A., Marzioch M., Clare J.J., Cousens D.J., Paddon C., Plumpton C. (2000). Functional coupling of mammalian receptors to the yeast mating pathway using novel yeast/mammalian G protein alpha-subunit chimeras. Yeast.

[84] Dowell S.J., Brown A.J. (2009). Yeast assays for G protein-coupled receptors. Methods Mol. Biol..

[85] Beukers M.W., van Oppenraaij J., van der Hoorn P.P., Blad C.C., den Dulk H., Brouwer J., AP I.J. (2004). Random mutagenesis of the human adenosine A2B receptor followed by growth selection in yeast. Identification of constitutively active and gain of function mutations. Mol. Pharmacol..

[86] Peeters M.C., Wisse L.E., Dinaj A., Vroling B., Vriend G., Ijzerman A.P. (2012). The role of the second and third extracellular loops of the adenosine A1 receptor in activation and allosteric modulation. Biochem. Pharmacol..

