# Supplementary material for: Molecular Effects of Auto-Antibodies on Angiotensin II Type 1 Receptor Signaling and Cell Proliferation
Source: Int J Mol Sci. 2022 Apr 2;23(7):3984. doi: 10.3390/ijms23073984 (PMC8999261; doi:10.3390/ijms23073984)
Supplement: Supplementary file 1 [file ijms-23-03984-s001.zip › ijms-1653607-supplementary.pdf]

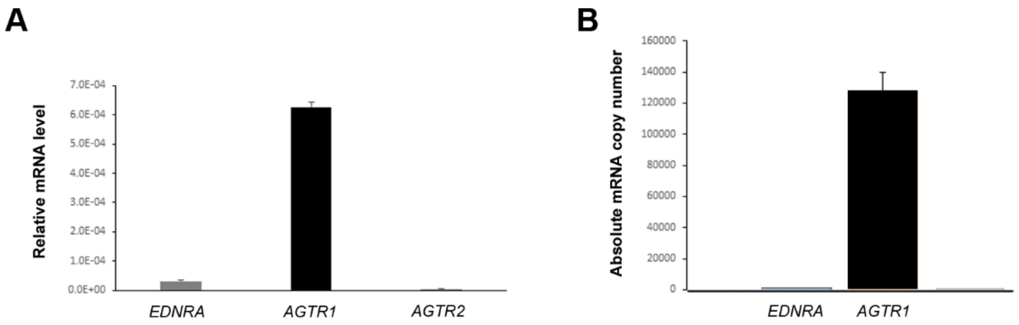

Suppl. Figure S1: AGTR1 is higher expressed than EDNRA and AGTR2. *(A) Relative mRNA level of EDNRA, AGTR1 and AGTR2, relative to β2microglobulin (HMEC-1), n=3 experiments; (B) Absolute mRNA copy number of EDNRA and AGTR1 (HMEC-1), n=2 experiments.*
